# EGFR-Targeted and MMP-Activated Membranolytic Peptides Kill Cancer Cells Specifically In Vitro and Reduce Tumor Growth In Vivo

**DOI:** 10.1021/acs.molpharmaceut.5c01774

**Published:** 2026-04-01

**Authors:** Arindam Pramanik, Andrew Booth, Dagmara Kobza, William J. Brackenbury, Simon D. Connell, Paul A. Beales, Thomas A. Hughes

**Affiliations:** † School of Medicine, 4468 University of Leeds, Leeds LS9 7TF, U.K.; ‡ Amity Institute of Biotechnology, Amity University, Noida, Uttar Pradesh 201303, India; § School of Chemistry and Astbury Centre for Structural Molecular Biology, 4468 University of Leeds, Leeds LS2 9JT, U.K.; ∥ Jack Birch Cancer Research Unit, York Biomedical Research Institute, Department of Biology, University of York, York YO10 5DD, U.K.; ⊥ School of Physics and Astronomy and Astbury Centre for Structural Molecular Biology, 4468 University of Leeds, Leeds LS2 9JT, U.K.; # School of Science, Technology and Health, 41872 York St John University, York YO31 7EX, U.K.

**Keywords:** membranolytic, cancer targeting, precision medicine, EGFR, matrix metalloproteinases

## Abstract

Membranolytic peptides are potential cancer therapeutics, although targeting cancer cells specifically remains an unmet challenge. We have modified the membranolytic peptide MP1, from *Polybia paulista*, to direct its action specifically to some cancer cells, thereby improving its cancer therapeutic characteristics and reducing its nonspecific toxicity. MP1 was modified by addition of sequences allowing binding to the cancer biomarker EGFR, with or without sequences directing cleavage by the cancer biomarker MMP-2. Toxicity was assessed in human breast cell lines and was correlated with EGFR expression and MMP-2 activity. Efficacy as an antitumor agent was assessed in MDA-MB-468 xenograft models. C-terminal addition of targeting sequences generally reduced cellular toxicities of peptides relative to wildtype MP1. Cell lines that retained the highest sensitivities to these fusion peptides expressed the highest EGFR and/or MMP-2 levels, supporting specific cytotoxic activity directed to these biomarkers. Treatment with an MMP-2 inhibitor significantly reduced the cell-killing activity of peptides containing MMP-2 cleavage sites, further supporting specific targeting. Fusion peptides significantly induced apoptosis and reduced survival in EGFR/MMP-2 high cancer cells, while sparing EGFR/MMP-2 low cells in standard tissue culture and 3D-spheroids. Systemic treatment with the EGFR-MMP-MP1 fusion significantly reduced tumor size in MDA-MB-468 xenograft models, confirming in vivo efficacy against cancer cells and acceptable systemic toxicity. We conclude that EGFR-MMP-MP1 peptides represent a novel cancer therapeutic for further development.

## Graphical abstract

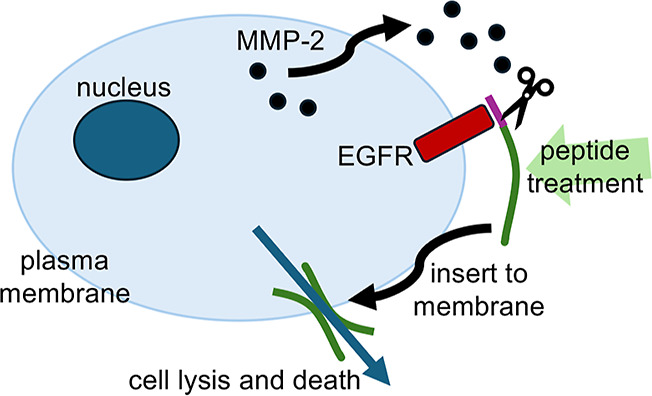

## Introduction

The majority of cancer therapeutics directly or indirectly target the same cancer property, namely, aberrant growth. This is the main mechanism of action for essentially all cytotoxic chemotherapies,^1^ which inhibit cell cycle processes, and many small molecule inhibitors^2^ and biologics,^3^ which typically target proteins that promote growth. Although these therapies have been successful, newer developments have increasingly focused on alternative mechanisms of action. A potential alternative therapeutic strategy is to lyse plasma membranes of cancer cells specifically, targeting the differences in lipid composition,^4^ membrane structure,^5^ and protein expression^6^ that cancer membranes display compared to normal membranes. Such lysis would potentially have anticancer activity both by directly reducing cancer cell survival and by inducing immune recognition of cancers through necrotic release of tumor antigens.^7^ Much of the work in this area has focused on harnessing naturally occurring membranolytic peptides, sourced from venoms and toxic secretions from a variety of organisms.^8^ However, despite development and testing of many different peptides, none have yet entered routine use in the clinic, although some clinical trials are ongoing.^8^ Surprisingly, many membranolytic peptides have been reported to exhibit intrinsic cancer-specific activity,^9,10^ although these reports have not always been confirmed in more extensive studies and this represents a key limitation for clinical translation as cancer therapies. An example is the peptide MP1 from the wasp *Polybia paulista*; this was initially reported as having some degree of cancer-specific lytic activity,^11,12^ although our recent work has failed to support this when using a larger panel of cancer and noncancer cell types.^13^ Nevertheless, the potential of these highly toxic lytic peptides as oncological therapies remains, if they can be directed specifically to cancer membranes.

In this work, we have targeted the activity of *Polybia paulista* MP1 to cancer cells by adding two different functionalities to its sequence. First, we have taken advantage of a peptide designed to bind specifically to the extracellular domain of the epidermal growth factor receptor (EGFR).^14^ EGFR is overexpressed in a range of common cancers, including breast,^15,16^ colorectal,^17,18^ and lung,^19^ and is well-established as a target for therapeutic inhibition^20^ and a surface biomarker to direct binding of therapeutics to cancer cells.^21–23^ Second, we have separated the EGFR-binding peptide from MP1 using a sequence that is cleaved by matrix metalloproteinase 2 (MMP-2).^24^ MMP-2 is frequently overexpressed in cancers, including in breast and colorectal, and expression is often associated with aggressive features and with poor survival.^25–27^ Accordingly, MMP-cleavage sites have been used to confer cancer-specific activation properties on potential therapeutics.^24,28,29^ We demonstrate that these additions combine to generate a fusion peptide that directs the lytic activity of MP1 to breast cancer cells expressing both targeted biomarkers (see graphic abstract). The resulting therapeutic peptide has low nonspecific toxicity so it can be delivered systemically and targets cancer cells effectively so as to reduce tumor growth in vivo. Thereby, we report a template for further development of a completely novel class of membranolytic cancer therapeutics.

## Materials and Methods

### Materials

Peptides were synthesized by Bio-Synthesis (Lewisville, TX, USA) to >95% purity, with counterion exchange to HCl. Quality control was conducted by HPLC and mass spectrometry by Bio-Synthesis, and we checked selected peptides for purity by analytical HPLC (see Figure S1). Peptides were lyophilized and shipped on dry ice. DOPC (1,2-dioleoyl-*sn*-glycero-3-phosphocholine) was obtained from Avanti Polar Lipids (Alabaster, AL, USA). NaCl and HEPES buffer were obtained from Sigma-Aldrich (St. Louis, MA, USA).

### Cell Lines

MDA-MB-468, MDA-MB-231, BT-474, AU-565, HB2, and MCF-10A cells were procured from American Type Culture Collection (ATCC) and validated for lack of mycoplasma contamination (MycoAlert; Lonza, Basel, Switzerland) and identity (short tandem repeat profiling; Source Bioscience, Nottingham, UK). Cells (except MCF-10A) were cultured in Dulbecco’s modified Eagle medium (DMEM) supplemented with 10% (v/v) fetal calf serum (FCS) and 100 units/mL penicillin–streptomycin (Thermo Scientific; Waltham, MA, USA). MCF-10A was cultured in DMEM supplemented with 5% horse serum, 0.1 μg/mL cholera toxin, 0.5 μg/mL hydrocortisone, 0.02 μg/mL epidermal growth factor, and 100 units/mL penicillin–streptomycin (Thermo Scientific, Waltham, MA, USA). Cells were grown at 37 °C in a humidified incubator with 5% CO_2_. Cells were maintained and experiments were performed under conditions ensuring cell densities that supported exponential growth.

### Cell Survival Assays

Cells were seeded in 96-well plates at 1 × 10^4^ cells/well in complete growth media and incubated for 18 h. Cells were then treated with peptides (0–250 μM) for up to 24 h. Cells were then incubated with 0.5 mg/mL MTT dissolved in PBS for 3 h. Formazan crystals were dissolved using 500 μL of isopropanol and absorbance was measured at 570 nm on a microplate spectrophotometer (Biotech Instruments, USA). For spheroids assays, MDA-MB-468 or HB2 cells (1000 cells/well) were seeded in 250 μL of DMEM supplemented with 10% FCS and 2.5% Matrigel (Corning, New York, USA) in low-adherent, round-bottom 96-well plates (Corning, New York, USA). The plates were centrifuged at 360*g* for 10 min and subsequently incubated for 48 h to allow spheroid formation. MDA-MB-468 or HB2 spheroids were then treated with peptides at the IC_50_ doses determined for MDA-MB-468 cells for 24 h. Cellular viability within spheroids was assessed by staining with Hoechst 33342 (5 μg/mL) for 30 min, followed by propidium iodide (PI) (1.5 μg/mL) for 10 min. Red fluorescence indicated nonviable cells stained with PI, whereas blue fluorescence represented both viable and nonviable cells stained with Hoechst 33342. The survival of spheroids was determined by calculating ratios of blue/red fluorescence using ImageJ software.

### Western Blotting

Cells were seeded into 6-well plates at 3 × 10^5^ and were cultured for 24 h. Western blots were performed as previously described.^30^ In brief, cells were lysed in RIPA buffer (Thermo Scientific, USA) and protein concentrations were determined using BCA assays (Merck, USA). Proteins were resolved by electrophoresis on 4–12% polyacrylamide gels (Bio-Rad, USA) and were transferred to PVDF membranes. Membranes were probed with EGFR or β-actin primary antibody (1:1000; Cell Signaling Technology, USA) overnight at 4 °C and secondary antibodies (1:5000; Cell Signaling Technology, USA) for 2 h. Bands were visualized with Pierce ECL (Thermo Scientific, USA), quantified via Chemi-doc (Bio-Rad, USA), and analyzed using ImageJ (NIH, USA).

### Large Unilamellar Vesicle Leakage Assays

These assays were performed exactly as previously described.^13^ In brief, DOPC solution (1 mL, 15 mM) was dried under nitrogen, to a thin film, further dried under vacuum, and rehydrated in 5(6)-carboxyfluorescein (CF) solution (120 mM in 10 mM HEPES pH 7.4) followed by 5 freeze–thaw cycles in liquid nitrogen. The suspension was then extruded through a 400 nm polycarbonate membrane and unencapsulated CF was removed by size exclusion chromatography. Leakage assays were carried out using a Hamilton Microlab Star M liquid handling robot (Hamilton Robotics) using a serial dilution of peptide into 10 mM HEPES (pH 7.4), followed by a fixed concentration of CF-loaded vesicles. Negative and positive controls were established by addition of vesicles to peptide-free buffer and buffer containing 0.6 mM Triton X-100. The final assay plate (384-well black OptiPlate, PerkinElmer LAS) was transferred to a PerkinElmer Envision plate reader where the CF fluorescence intensity was measured (ex: 495 nm; em: 517 nm).

### MMP-2 Analyses

Cells were seeded in 6-well plates at 3 × 10^5^ in complete medium and allowed to grow for 48 h. 500 μL of cell media from each cell line was collected and centrifuged at 5000 rpm for 15 min at 4 °C. Supernatants were collected and 50 μL of each was added in 3 replicate wells of a monoclonal mouse antihuman MMP-2 antibody precoated 96-well ELISA plate (Human Total MMP-2 kit; BioLegend, USA). It was then mixed with the respective assay buffers as per the kit instruction. The plate was then sealed and incubated at room temperature (2 h). The supernatant sample was then discarded and the wells were washed with assay buffer several times. Next, 100 μL of Human Total MMP-2 Detection Antibody solution was added into the wells and incubated (1 h). 100 μL of Avidin-HRP solution was then added for 1 h, and finally, after washing, 100 μL of substrate solution was added. Absorbance was measured at 450 nm (Biotech Instruments, USA). The final MMP-2 concentration was calculated against a standard curve. For inhibitor studies, MDA-MB-468 cells were seeded in 96-well plates at 1 × 10^4^ cells/well in complete growth media and incubated for 18 h. Cells were then treated with MMP-2 inhibitor (2 μM chlorhexidine dihydrochloride; Santa Cruz Biotechnology, USA), peptide (20 μM EGFR-MP1 or 30 μM EGFR-MMP-MP1), or the combination for 24 h. Post-treatment, MTT assays were performed as detailed above.

### Apoptosis Analysis Using Flow Cytometry

Apoptosis was studied using Annexin V/PI assays and flow cytometry. In brief, MDA-MB-468 and HB2 cells were seeded in 6-well plates at 3 × 10^5^ in complete medium and allowed to grow for 18 h. Cells were then treated with the IC_50_ doses for MDA-MB-468 cells of EGFR-MP1, EGFR-MMP-MP1, or EGFR-MMP-D2K for 24 h. Following treatment, the cells were scraped from the plate and washed with Annexin binding buffer. Then 2 μg/mL Annexin V-FITC (Thermo Fisher Scientific, Waltham, MA, USA) was added and incubated for 15 min in the dark. Cells were then washed and 1 μg/mL PI (Thermo Fisher Scientific, Waltham, MA, USA) was added. Then cells were analyzed using a CytoFLEX S flow cytometer (Beckman Coulter, UK), and the data were processed using FlowJo (v10.6.1).

### Ex Vivo Hemolysis Assay

Blood was collected from five 10-week-old male C57BL/6 mice via cardiac puncture following cervical dislocation, using pediatric blood collection tubes containing K3 EDTA (Greiner; Slušovice, Czechia). Blood was centrifuged (500*g*; 5 min; 4 °C) and plasma was removed. Red blood cells (RBCs) were resuspended to their original volume using 150 mM NaCl. RBCs were repeatedly washed and diluted to 1:50 in PBS. For a positive control, 1% (v/v) Triton X-100 was used (100% lysis), while cells incubated in PBS served as a negative control (0% lysis). 200 μL of RBCs in triplicate was treated with peptides and incubated on an orbital shaker (100 rpm; 1 h; 37 °C). Samples were centrifuged (500*g*; 5 min) and supernatants, containing lysed RBCs, were collected. Hemolysis was quantified by measuring absorbance at 540 nm.

### In Vivo Studies

These were performed by HD Biosciences (Shanghai, China). Procedures related to handling, care, and treatment of animals were performed according to guidelines approved by the HD Biosciences Institutional Animal Care and Use Committee (reference number AUC105), under the criteria of the Association for Assessment and Accreditation of Laboratory Animal Care (AAALAC). Protocols and AAALAC accreditation were reviewed by the Animal Welfare and Ethical Review Committee at the University of Leeds and were approved (reference number THAWERC222707; date 28/7/2022). Protocols and data are reported in accordance with the ARRIVE guidelines (https://arriveguidelines.org/arrive-guidelines). Female NCG mice (6–8 weeks old) were purchased from GemPharmatech Ltd. (Nanjing, China). Xenografts were established by subcutaneously injecting exponentially growing MDA-MB-468 cells (1 × 10^7^ cells in 0.2 mL in D-PBS 1:1 with Matrigel) into the right flank of the mice. Once tumor volumes reached 150–200 mm^3^, mice bearing xenografts were assigned into two groups using stratified randomization based upon tumor volumes in Microsoft Excel to ensure groups were comparable at baseline. Two groups were used: control group (13 animals) and test group (7 animals); group sizes were not determined by power calculation due to lack of data on which this could be based. The uneven group sizes were due to limited availability of EGFR-MMP-MP1 peptide. The test group was administered three intravenous doses via tail vein injection of 500 μg of EGFR-MMP-MP1 on days 1, 3, and 5, while the control group received equivalent volumes of saline. Tumor volumes were monitored using calipers, and body weight was monitored throughout the experiment; no animals were excluded. Experimenters were not blinded to the treatment group. The experiment was terminated on day 27 and the tumor tissue was extracted and weighed.

### Statistics, Online Data, and Data Availability Statement

In vitro experiments were performed as biological triplicates unless stated otherwise in figure legends. Statistical analyses, as described in figure legends, were performed using Prism v10 (GraphPad; Boston, MA, USA). The DepMap portal data were accessed at https://depmap.org/portal/gene/EGFR?tab=dependency&characterization=expression as transcripts per million averaged across replicates of each sample. The Protein Atlas portal data were accessed at https://www.proteinatlas.org/ENSG00000146648-EGFR/cell+line#breast_cancer as normalized transcripts per million. Essentially, all data are included within the figures, although primary data can be requested from the authors without restriction.

## Results

### MP1 Toxicity and Cell-Line Specificity Can Be Modified Using C-Terminal Extensions

We previously reported that MP1 is a membranolytic peptide capable of causing cell death in human cells with IC_50_ doses varying across a panel of cell lines by over 6-fold without specificity for cancer cells^13^ (Figure S2). Our first aim was to assess whether MP1 could gain cancer specificity by extending the peptide sequence with additions to induce binding to the surface of cancer cells or to allow activation by cancer cells. We investigated addition of a targeting sequence that directs binding to EGFR.^14^ Also, we linked this to MP1 either by a simple GG linker or using a proteolytic cleavage site that can be targeted by MMP-2.^24^ We attached these targeting or cleavage sequences to either the N-terminus or the C-terminus of MP1. See Table 1 for sequences used and our nomenclature for the peptides.

**Table 1:** Peptide Sequence Details^a^

peptide name	peptide sequence (N- to C-terminus)
MP1	**IDWKKLLDAAKQIL**
N-EGFR-MP1	*YHWYGYTPENVI*GG**IDWKKLLDAAKQIL**
N-EGFR-MMP-MP1	*YHWYGYTPENVI*GPLGIAGQ**IDWKKLLDAAKQIL**
EGFR-MP1	**IDWKKLLDAAKQIL**GG*YHWYGYTPENVI*
EGFR-MMP-MP1	**IDWKKLLDAAKQIL**GPLGIAGQ*YHWYGYTPENVI*
EGFR-D2K	**I** K **WKKLLDAAKQIL**GG*YHWYGYTPENVI*
EGFR-MMP-D2K	**I** K **WKKLLDAAKQIL**GPLGIAGQ*YHWYGYTPENVI*

a The MP1 sequence is in bold; substitutions are underlined; the EGFR-targeting sequence is in italics.

A panel of human breast epithelial cell lines was established, including two lines from a nontransformed origin (MCF-10A; HB2) and four cancer lines (BT-474; AU-565; MDA-MB-231; MDA-MB-468). Cells were treated with different doses of MP1 or the modified peptides. Survival was assessed relative to untreated using MTT assays (Figures 1 and S2) and IC_50_ doses were determined for each peptide in each cell line.

**Figure 1:**
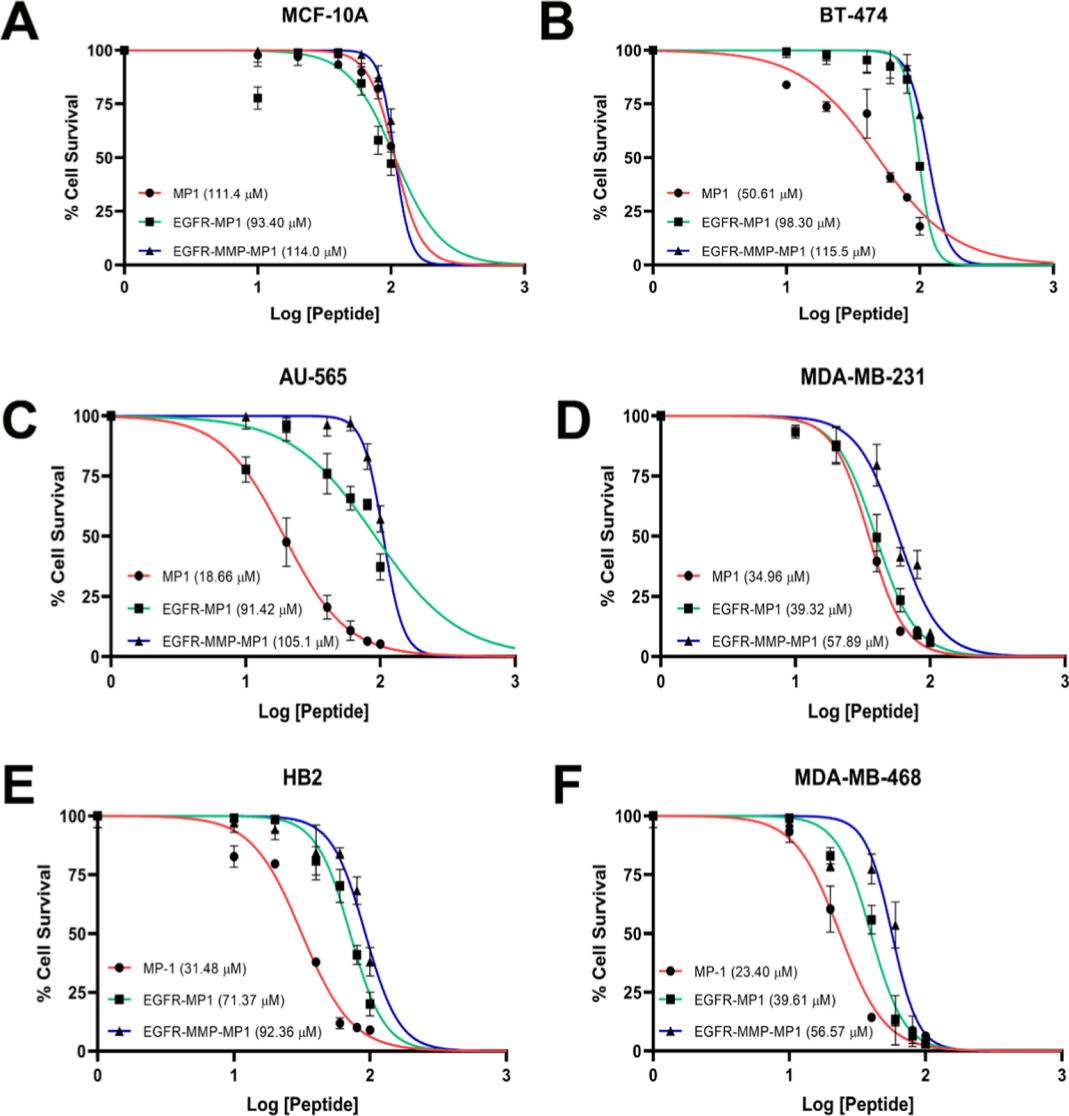
Addition of EGFR-targeting and/or MMP-2 cleavage sequences to MP1 modifies lytic activity in a cell-specific manner. Cell lines as marked were treated with a range of doses of MP1, EGFR-MP1, or EGFR-MMP-MP1 for 24 h and cell survival was assessed using MTT assays. Survival is shown relative to untreated control and IC_50_ values were extracted from best-fit curves. Data represent means and standard errors of three independent experiments.

We found that N-terminal fusions had very limited activity, with IC_50_ doses estimated to be up to 10-times higher when compared to MP1 itself (Figure S3). We concluded that an unmodified N-terminus was required for MP1 function and that these fusions had little potential as anticancer agents; these were not investigated further. By contrast, the C-terminal fusions showed effects that varied according to the cell line (Figure 1). In most lines, EGFR-targeting reduced efficacy, although the extent of reduction varied from negligible (MDA-MB-231) to 5-fold (AU-565). Similarly, addition of the MMP cleavage site further reduced efficacy. MCF-10A cells, however, behaved differently; in this cell line, neither fusion peptide showed strikingly different efficacy when compared to MP1 (Figure 1A), although it should be noted that this cell line is especially resistant to the wildtype MP1 peptide and therefore further reductions in function in an already poorly functional peptide may be difficult to detect. We concluded that C-terminal fusion peptides demonstrated activities that were dependent on the characteristics of the cells used and therefore that their dependence on EGFR expression and MMP activity should be investigated.

We also assessed the activity of MP1 and EGFR-MMP-MP1 against simple model membrane vesicles, to demonstrate that the peptides act directly on membranes in a system where downstream biology cannot be induced. Vesicles were assembled using the lipid DOPC and were loaded with the carboxyfluorescein at concentrations that are fluorescence-self-quenching. Vesicles were treated with a range of peptide concentrations, and fluorescence resulting from release of carboxyfluorescein and consequent loss of quenching was quantified (Figure S4). As expected, MP1 was highly effective at lysing vesicles, which can be quantified using the concentration required to achieve 50% of maximal fluorescence: 75 nM. In comparison, EGFR-MMP-MP1 showed a very large reduction in efficacy (more than 18-fold; 50% lysis concentration 1.39 μM), which reflects the complete lack of EGFR and MMP-2 in this purified system.

### Activity of the Targeted and Cleavable Peptides Varies with Expression of EGFR and MMP-2

To correlate cytotoxic efficacy of fusion peptides with EGFR expression, we quantified EGFR in the cell lines using Western blots (Figure 2A). The cell lines divided broadly into two groups: MDA-MB-468 and MDA-MB-231 cells, had similar, relatively high, expression levels while the remaining lines had low levels. RNA expression data for the cancer lines, available from two independent resources (DepMap Portal and Human Protein Atlas), also confirmed the highest EGFR expression in MDA-MB-468 cells, followed by MDA-MB-231 cells (Figure S5). In accordance with their high EGFR expression, these two lines showed the greatest sensitivity to EGFR-MP1 across the cell line panel (Figure 1) and also showed comparatively small reductions in efficacy associated with the addition of EGFR-targeting when compared to wildtype MP1 itself. This was in contrast to, for example, AU-565 that had the greatest intrinsic sensitivity to wildtype MP1 but showed striking and substantial reductions in efficacy from EGFR-targeting in accordance with its low EGFR expression.

**Figure 2:**
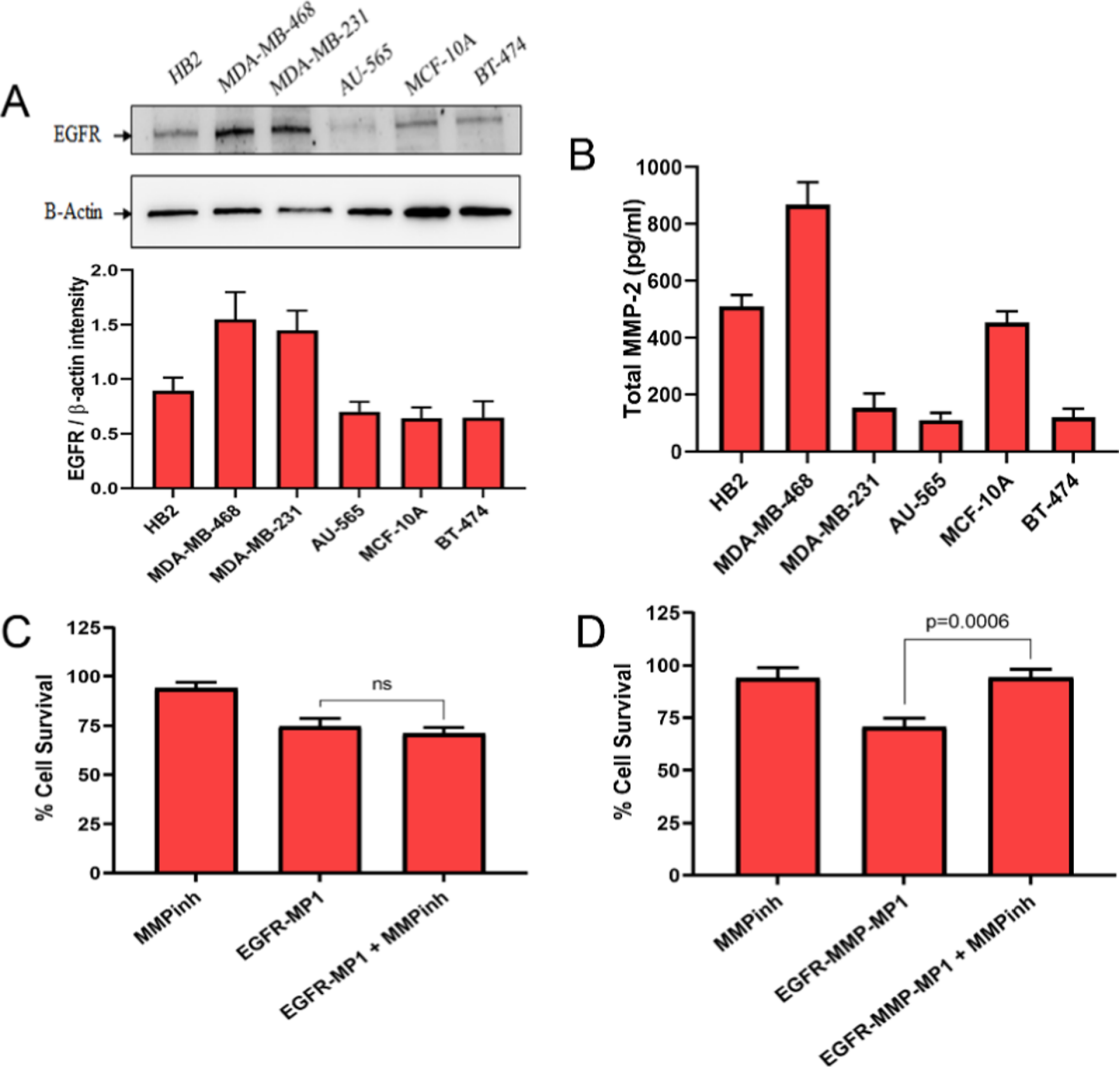
MDA-MB-468 cells express high levels of EGFR and MMP-2 and show MMP-2-dependent activity of EGFR-MMP-MP1. (A) EGFR expression in breast epithelial cell lines was assessed using Western blots (top) and expression was quantified relative to actin using densitometry (bottom). (B) Soluble MMP-2 was quantified in the medium of cultured cells by ELISA. (C,D) MDA-MB-468 cells were treated with 20 μM EGFR-MP1 (C) or 30 μM EGFR-MMP-MP1 (D) in the presence or absence of 2 μM of the MMP-2 inhibitor chlorhexidine dihydrochloride (MMPinh) or with the inhibitor alone. Cell survival was measured using MTT assays and is shown relative to an untreated control. Quantitative data represent means and standard errors of three independent experiments. Statistical analyses were performed using a one-way Student’s *t*-test.

Next, we quantified expression of MMP-2 in the cell lines using ELISAs (Figure 2B). MDA-MB-468 demonstrated the highest secreted concentration, which was more than 4-fold higher than the remaining cancer lines, with the two nontransformed lines (HB2; MCF-10A) showing intermediate activities. Accordingly, MDA-MB-468 cells showed relatively high sensitivity to the targeted and cleavable peptide EGFR-MMP-MP1, as—surprisingly—did MDA-MB-231 cells (Figure 1) despite relatively low MMP-2 activity. AU-565 cells had the lowest MMP-2 expression (Figure 2B) and also showed the greatest loss of efficacy associated with the addition of the MMP cleavage site, indicating that MP1’s efficacy was suppressed in this fusion when MMP-2 levels were low.

We were also interested to test formally whether the efficacy of EGFR-MMP-MP1 was dependent on MMP-2 activity. Therefore, we treated MDA-MB-468 cells with EGFR-MMP-MP1 or with EGFR-MP1, in the presence or absence of the MMP-2 inhibitor chlorhexidine dihydrochloride, and assessed cell survival relative to untreated (Figure 2C,D). Treatment with chlorhexidine dihydrochloride alone caused a small and nonsignificant decrease in cell survival, while—as expected—both peptides reduced cell survival significantly. Treatment with chlorhexidine dihydrochloride completely halted the cell death induced by EGFR-MMP-MP1 (Figure 2D; *p* < 0.001) while it had no effect on the activity of EGFR-MP1 (Figure 2C), thereby demonstrating that the MMP-2 cleavage site confers activity on the peptide that can be blocked by the MMP-2 inhibitor.

We concluded that efficacy of fusion peptides correlated with expression or activity of the markers they were designed to target, with MDA-MB-468 cells showing particularly favorable characteristics for successful targeting by this combination.

### Inclusion of EGFR-Targeting and MMP-Cleavage Sequences Confers a Therapeutic Window to Target EGFR- and MMP-2-Positive Cells

Next, we aimed to assess whether EGFR-targeting and MMP-cleavage sequences could give sufficient specificity to our peptides to kill target cells (cancer cells that are EGFR- and MMP-2-positive) while sparing nontarget cells (cells with low expression of both or either marker). This analysis is potentially confounded by differences in intrinsic sensitivity to wildtype MP1, highlighted, for example, by the relative resistance to all MP1-derived peptides seen in MCF-10A and BT-474 cells that was unrelated to EGFR or MMP expression. We selected MDA-MB-468 cells as our ideal target cell since it had the highest expression of both EGFR and MMP-2 (Figure 2). For comparison, we selected HB2 cells as a representative nontarget cell since these cells uniquely had intrinsic sensitivity to wildtype MP1 that was similar to MDA-MB-468 cells (HB2 IC_50_ 31 μM, compared to MDA-MB-468 23 μM; Figure 1) but also had lower expression of both targeting markers (see Figure 2).

HB2 or MDA-MB-468 cells, in either standard 2D culture or cultured as 3D spheroids, were treated with IC_50_ doses of either EGFR-MP1 or EGFR-MMP-MP1 as determined in Figure 1 for MDA-MB-468 cells or were treated with control. Cell death in these cultures was assessed in the 2D cultures by Annexin-V/PI staining (Figure 3A; Figure S6 for representative cytometry plots). Cell survival in spheroids was assessed by Hoechst 33342/PI staining and microscopy (Figure 3B; Figure S7 for representative microscopy images). We found that both peptides induced substantial apoptosis and reduced cell survival in MDA-MB-468 cells, while effects on HB2 cells were negligible. This finding was compatible with the favorable ‘selectivity index’ for EGFR-MMP-MP1 between the two cell lines, calculated as the ratio of the IC_50_ dose for the noncancer line to the IC_50_ dose of the cancer line^31^ (IC_50_ doses shown in Figure 1: selectivity index, 1.63). We concluded that our EGFR-targeting and MMP-activation strategy was sufficient to allow specific killing of target cells.

**Figure 3:**
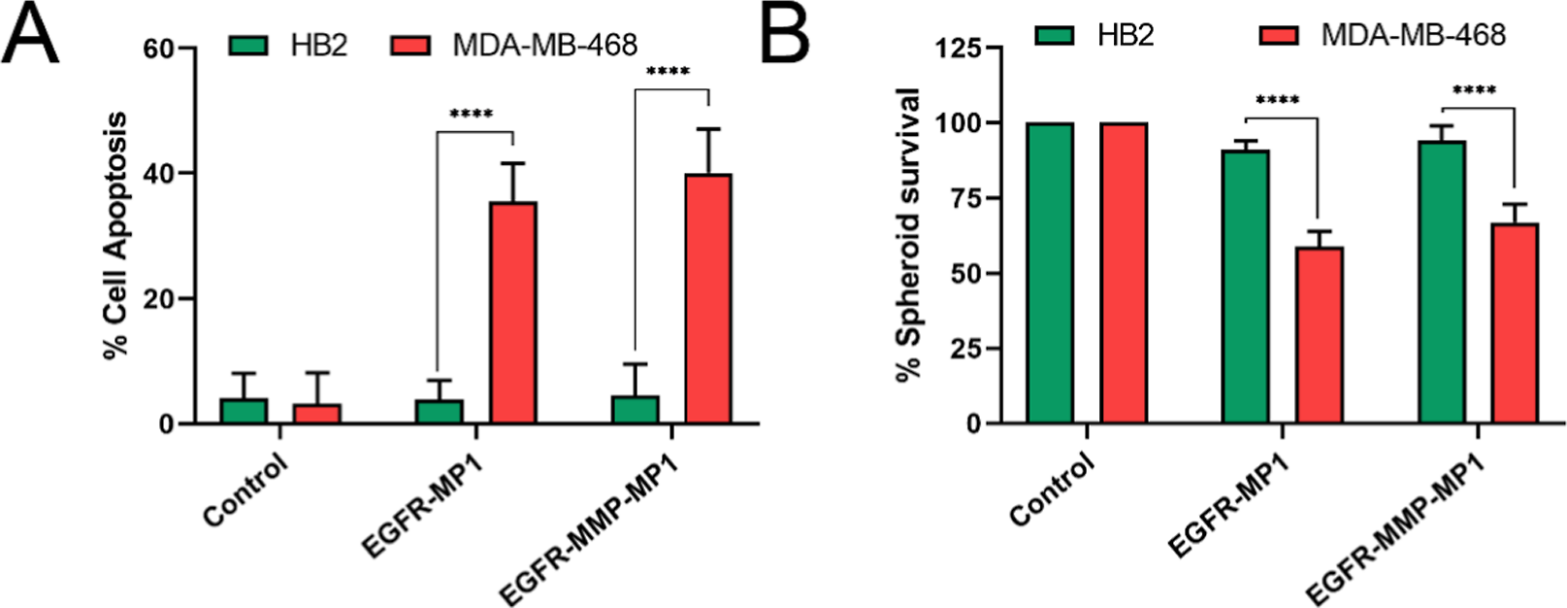
Directing MP1 to EGFR and MMP-2 allows targeting of MDA-MB-468 cancer cells, sparing noncancerous HB2 cells. (A) HB2 or MDA-MB-468 cells in 2D culture were treated with EGFR-MP1 (39.6 μM) or EGFR-MMP-MP1 (56.6 μM) for 24 h. Apoptosis was quantified using Annexin V/PI staining and flow cytometry. (B) 3D spheroids were established with HB2 or MDA-MB-468 cells, and these were treated with EGFR-MP1 (39.6 μM) or EGFR-MMP-MP1 (56.6 μM) for 24 h. Cell survival was quantified by counting fluorescent cells after staining with PI/Hoechst 33342 by fluorescence microscopy. Data represents means and standard errors of three independent experiments, and statistical analyses were performed using two-way ANOVA tests (**** indicates *p* < 0.0001).

### Substitutions within the MP1 Sequence Can Increase Toxicity, although without Improving Specificity for Cancer Cells

We have previously reported on changes in MP1 activity caused by substitution of individual residues within its sequence.^13^ We were now interested to assess whether any of these substitutions could improve efficacy and/or specificity of our fusion peptides. In Figure 4A, we present a reanalysis of our previous data^13^ demonstrating changes in IC_50_ values associated with four separate single residue substitutions. We show that in MDA-MB-468 cells, increased efficacy in terms of cell killing (i.e., reduced IC_50_ values) results from substitutions where the aspartic acid residues at position 2 or 8 are replaced with lysines (D2K and D8K, respectively). However, we noted that D8K also showed increased efficacy in MCF-10A cells, which for our current purposes represents a ‘nontarget’ cell line. Therefore, we selected D2K as the substitution with the most potential to improve efficacy and specificity.

**Figure 4:**
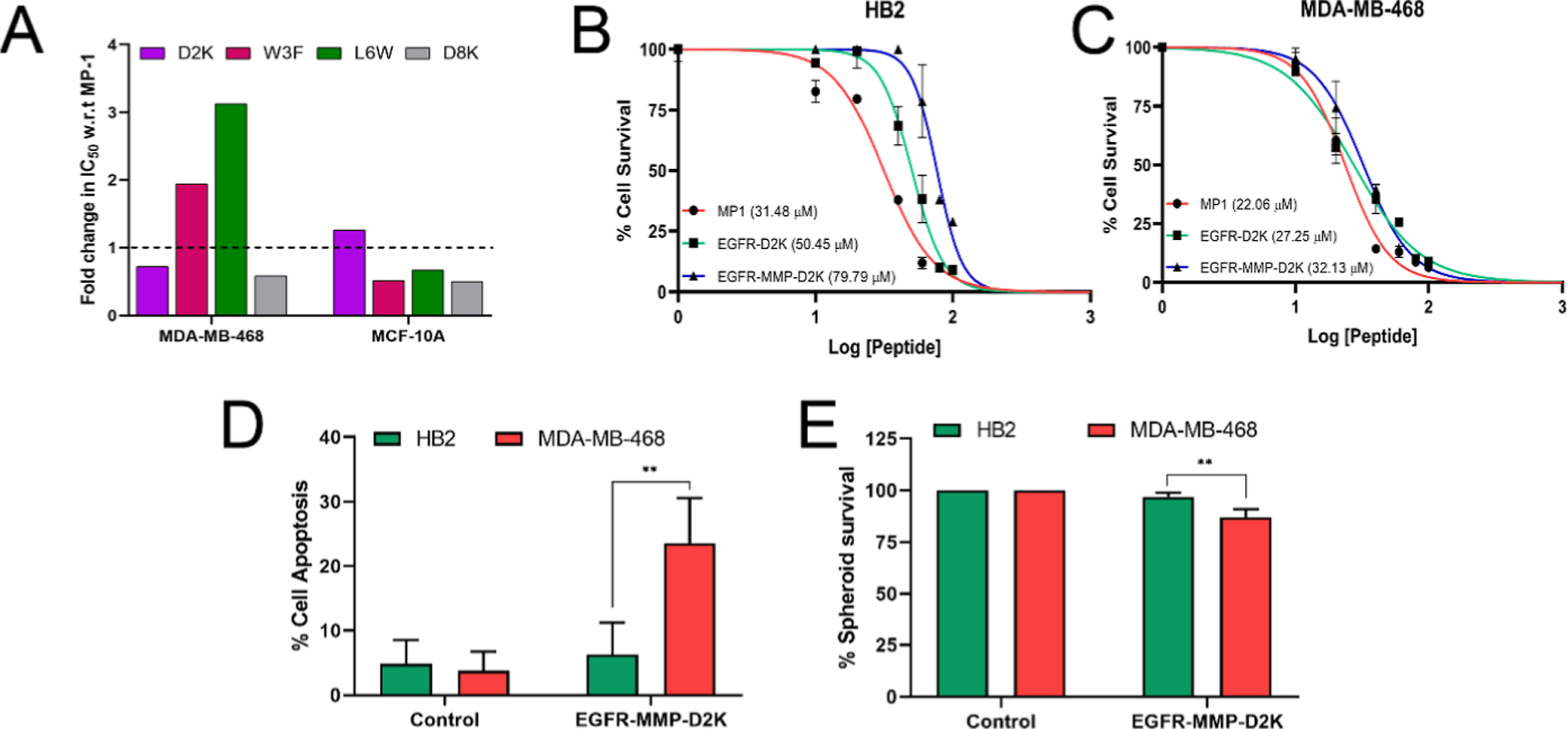
The D2K substitution in MP1 increases toxicity in the context of EGFR-MP1 and EGFR-MMP-MP1, while specificity for MDA-MB-468 cells is retained although not enhanced. (A) IC_50_ values for MP1 variants with single residue substitutions (D2K, W3F, L6W or D8K) were determined in MDA-MB-468 and MCF-10A cells using MTT assays (Booth et al 2025 ^13^). Data represent fold change in IC_50_ relative to MP1; variants below the dotted line have reduced IC_50_ values and are therefore have improved efficacy. (B and C) The D2K substitution was synthesised in the context of the fusion peptides, to create EGFR-D2K and EGFR-MMP-D2K. HB2 or MDA-MB-468 were treated for 24 h with various doses of EGFR-D2K or EGFR-MMP-D2K, or with MP1 for comparison, and cell survival was quantified using MTT assays relative to untreated. IC_50_ values were extracted from best-fit curves. (D and E) HB2 and MDA-MB-468 cells in 2D (D) or 3D spheroid (E) culture were treated with EGFR-MMP-D2K (32.1 μM) for 24 h and apoptosis (D) or cell survival (E) was analysed as in Figure 3. Data represents means and standard errors of three independent experiments, and statistical analyses were performed using two-way ANOVA tests (** indicates *p* < 0.01).

The D2K substitution was synthesized in the context of our EGFR- and EGFR-MMP-extended peptides to create EGFR-D2K and EGFR-MMP-D2K (see Table 1). HB2 or MDA-MB-468 cells, our established pair of nontarget and target cells, were treated with different doses of wildtype MP1 or with the targeted D2K peptides, and survival was assessed relative to untreated as previously done (Figure 4B and C). In both cell lines, the D2K peptides were substantially more toxic than their MP1 versions (compare to Figure 1E,F), with a mean reduction in IC_50_ of 17.5 μM. Disappointingly, there was no suggestion that increased toxicity was greater in the target cell line MDA-MB-468 as opposed to the nontarget HB2. Nevertheless, we repeated our assessment as in Figure 3 of whether the targeted peptide provided sufficient specificity to kill MDA-MB-468 cells while sparing HB2 cells (Figure 4D and E; Figures S8 and S9 for representative primary data). We found that EGFR-MMP-D2K significantly reduced cell survival in MDA-MB-468 cells while effects on HB2 cells were minimal, demonstrating a therapeutic window for this substituted peptide with increased overall efficacy.

### EGFR-MMP-MP1 Retards Tumor Growth In Vivo

Our next aim was to assess whether our various peptides had potential as anticancer therapeutics using an in vivo model. To our knowledge, MP1 or MP1-derived peptides have not previously been used experimentally in vivo, therefore we first performed an in vitro hemolysis assay to aid selection of peptides that could be suitable for intravenous delivery. Red blood cells were isolated from mouse blood and treated with three different doses of MP1 wildtype or our targeted peptides (EGFR and EGFR-MMP versions of both MP1 and D2K) and hemolysis was measured (Figure 5A). Wildtype MP1 caused unacceptably high levels of hemolysis at all doses, highlighting its relative lack of specificity in terms of cell types lysed. All the fusion peptides showed dramatically lower levels of hemolysis, in accordance with their expected targeting to cancer biomarkers. However, D2K variants showed higher levels of hemolysis than their matched MP1 versions at the two lowest doses used, rising to as high as 5.9% at 50 μM (EGFR-MMP-D2K). From the available data, we selected EGFR-MMP-MP1 for further investigation in vivo, as it demonstrated low hemolysis at relevant doses (Figure 5A) and showed strong efficacy against our target cell line MDA-MB-468 (Figure 3).

**Figure 5:**
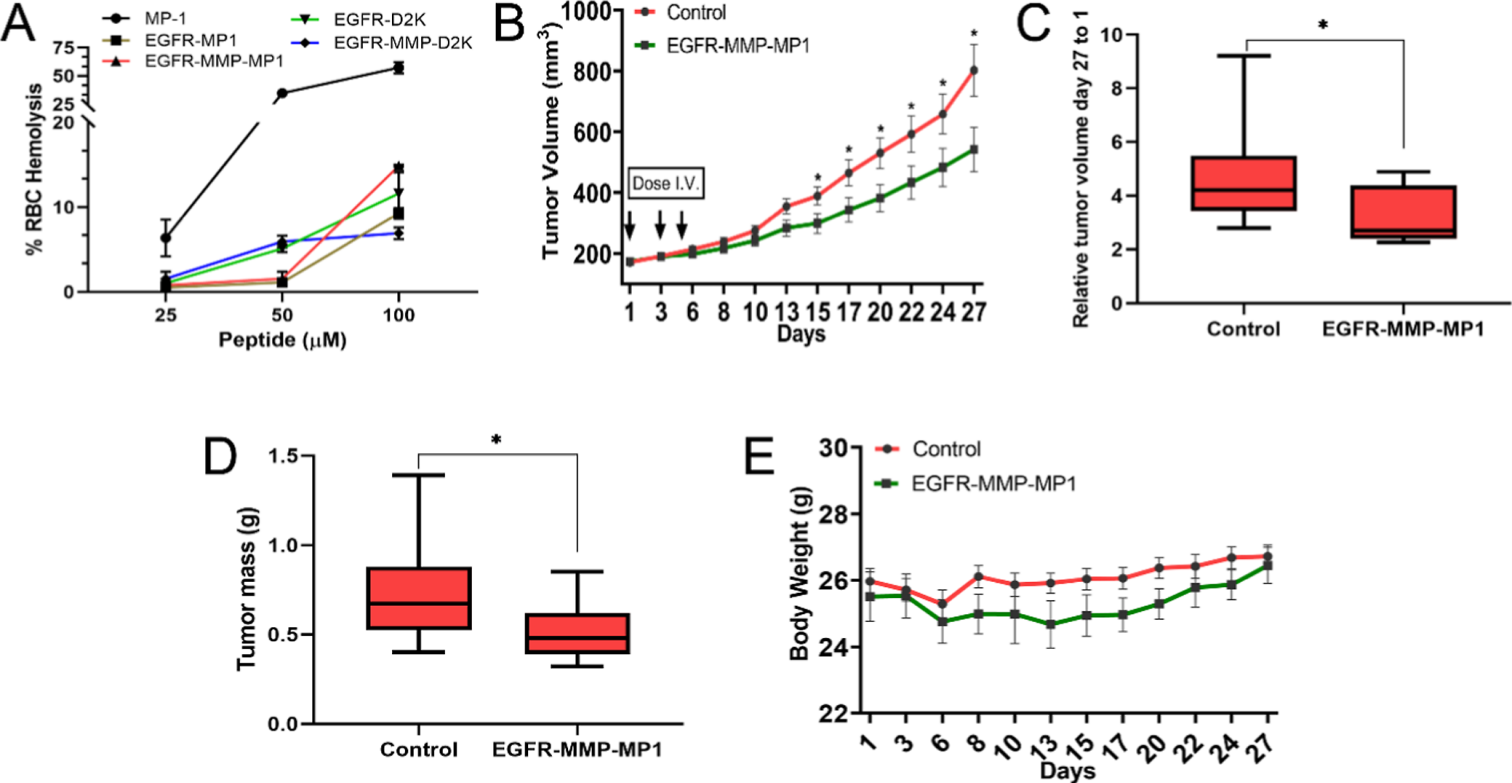
EGFR-MMP-MP1 shows effective anticancer activity on MDA-MB-468 xenografts in vivo. (A) Hemolysis assays were performed for 5 peptides sequences (MP1, EGFR-MP1, EGFR-MMP-MP1, EGFR-D2K, and EGFR-MMP-D2K) in fresh red blood cells isolated from mice. (B–E) MDA-MB-468 xenografts were established in NCG mice before treatment with three doses of 500 μg of EGFR-MMP-MP1 peptide or saline control as shown. Tumor size (C) and animal weight (E) were measured at 2–3 day intervals for a total of 27 days. Increase in tumor size over the course of the experiment was quantified as fold-change in size in each group (C). Tumor masses were measured after termination of the experiment (D). Data represent means with standard errors. Statistical analyses were performed using Mann–Whitney *U* tests (* indicates *p* < 0.05).

NCG immune-compromised female mice were implanted subcutaneously with MDA-MB-468 xenografts. Animals were randomized to control and treatment groups and were treated by tail vein injection with doses of control (saline) or 500 μg EGFR-MMP-MP1 on days 1, 3, and 5 of the experiment. Tumor size and animal weight were monitored every 2 to 3 days for a total of 27 days, after which animals were sacrificed and tumors were dissected and weighed. Treatment with EGFR-MMP-MP1 caused a significant and sustained retardation in tumor growth (Figure 5B–D). At the end of the experiment, the mean tumor volume of tumors treated with EGFR-MMP-MP1 was only 67.5% (*p* < 0.05) of the untreated tumors, and similarly, tumor mass was only 69.35% (*p* < 0.05). Overall tumor growth from day 1 to day 27 was a 3.1-fold increase in the EGFR-MMP-MP1 treated group, compared to 4.6-fold for the control group (Figure 5C; *p* < 0.05). Animal weights, as a surrogate for side-effects, were slightly reduced in the treatment group, although they recovered by the end of the experiment (Figure 5E); we take this to suggest that overall nonspecific toxicity was acceptable, although thorough assessments of other toxicological parameters including blood biochemistry and organ histology will be required to support this interpretation. We concluded that EGFR-MMP-MP1 is effective at killing target cancer cells and is potentially associated with acceptable nonspecific toxicity. This novel agent has potential as an anticancer therapeutic for EGFR-positive and MMP-2-positive cancers.

## Discussion

Membranolytic peptides have been studied extensively in vitro as potential anticancer therapeutics,^8–10^ with some going on to in vivo assessment^32,33^ or even early phase clinical trials.^34–36^ However, toxicity associated with activity against noncancer cells remains a substantial and mainly unaddressed problem. Investigators have attempted to limit these off-target effects to some extent through delivery by intratumoral injection^32–34,36^ rather than systemically. Despite this mitigation, agents have still shown substantial toxicity in mouse models.^32^ Nevertheless, the peptide LTX-315 entered clinical trials as a first-in-class untargeted membranolytic peptide delivered through intratumoral injection^34^ and has shown some evidence of antitumor activity and a toxicity profile that could be tolerable. Further trials of this, and at least two different membranolytic peptides have recently recruited or are underway.^35–37^ However, use of intratumoral injections presents challenges for integration into treatment of many cancers. This is because relatively few cancers are located to allow intratumoral injection, and also because the predominant curative regimen for many primary solid cancers prioritizes resection surgery, with further therapies usually in the adjuvant (postsurgical) setting. In any event, the main target of systemic therapies for primary solid cancers is often the subclinical disseminated cancer cells in unknown locations, which if left untreated can develop into distant metastatic recurrences; intratumoral injection is unable to substitute for this role. Consequently, systemic delivery of membranolytic peptides would maximize their potential utility. Critically, however, to allow systemic delivery, the issue of specificity to cancer cells needs to be addressed.

We have examined the membranolytic peptide MP1 from the wasp species *Polybia paulista*, which was reported to show intrinsic specificity to cancer cells.^11,12^ However, we failed to confirm this specificity, demonstrating its activity on human cells to be irrespective of cancer or noncancer origin (Figure S2). We also found it to have unacceptably high hemolytic activity (Figure 5A), ruling out systemic delivery. Therefore, we focused on extending the MP1 sequence to target it more effectively to cancer cells and spare nontarget cells. This approach is related to that taken with the peptide EP-100,^38^ which comprises an 18-residue lytic peptide linked directly to a 10-residue sequence that directs binding to a cancer biomarker, the GnRH receptor. This peptide showed lytic activity in vitro that was target-specific and accordingly was safely delivered systemically in mouse models.^39^ Human clinical trials are ongoing, using systemic delivery by intravenous infusion, and initial data show a good safety profile and some anticancer activity.^40^ In our work, we extended the MP1 lytic peptide to include sequences known to bind to EGFR, a very commonly expressed cancer biomarker,^20^ and to direct cleavage from this targeting sequence by MMP-2, a protease commonly upregulated in cancer cells.^29^ This strategy was designed to use biomarkers that are overexpressed across a wide range of cancer types including the three commonest solid cancers (for example, more widely than the GnRH receptor^41^) and to increase cancer-specificity further by having both targeting and proteolytic activation elements. We have focused on breast cancer, and we found that cell lines from the triple negative subclassification (MDA-MB-468 and MDA-MB-231) are the most targetable using this biomarker combination (Figures 1 and 2). This is ideal since triple negative primary breast cancer currently lacks molecularly targeted therapies, has the poorest outcomes, and is most in need of alternative approaches.^42^ Our data demonstrate that the peptide EGFR-MMP-MP1 is well-tolerated in systemic treatment and causes significant retardation of tumor growth in vivo (Figure 5). We believe this novel peptide has great potential for further preclinical and clinical development as a cancer therapeutic.

## Conclusion

We have designed and tested a novel cancer-targeted membranolytic peptide that can be delivered systemically in vivo with acceptable toxicity and causes significant retardation of tumor growth. Membranolytics are an exciting new class of potential cancer therapeutics with a mode of action that is completely different from all established cancer therapies. Technologies such as we describe to target the membranolytic activities effectively to cancer cells will be required to allow them to meet their potential as powerful additions to the range of therapeutic options to improve cancer outcomes.

## Supplementary Material



## Data Availability

Data are available within the manuscript/Supporting Information or can be requested directly from the communicating authors.

## Notes

Representative analytical HPLC chromatogram for MP1 (Figure S1); IC_50_ doses for MP1 in various cell lines (Figure S2); dose responses with N-terminal additions of EGFR- or MMP-targeting to MP1 (Figure S3); dose responses for MP1 and EGFR-MMP-MP1 in carboxyfluorescein-loaded unilamellar vesicles (Figure S4); relative EGFR expression levels from online data (Figure S5); representative dot plots for data shown in Figure 3A (Figure S6); representative immunofluorescence images for data shown in Figure 3B (Figure S7); representative dot plots for data shown in Figure 4D (Figure S8); and representative immunofluorescence images for data shown in Figure 4E (Figure S9) (PDF)
